# Comment on “do certain blood groups increase COVID-19 severity and mortality?”

**DOI:** 10.1016/j.gloepi.2026.100246

**Published:** 2026-01-17

**Authors:** Prashant Ramdas Kokiwar, Amit Singh Pawaiya, Ranjana Roy, Reenoo Jauhari

**Affiliations:** a Department of Community Medicine, Malla Reddy Institute of Medical Sciences, Malla Reddy Vishwavidyapeeth, Suraram, Hyderabad 500055, Telangana, India; b Department of Community Medicine, School of Medical Sciences and Research, Sharda University, Greater Noida, India; c Dr. D. Y. Patil Medical College, Hospital and Research Centre, Dr. D. Y. Patil Vidyapeeth (Deemed-to-be-University), Pimpri, Pune 411018, Maharashtra, India; d Faculty of Pharmaceutical Sciences, Graphic Era Hill University, Dehradun, India; e Centre for Promotion of Research, Graphic Era Deemed University, Dehradun, India

Dear Editor,

In the article “Do certain blood groups increase COVID-19 severity and mortality?” the authors report a retrospective cohort of 570 RT-PCR–confirmed adults with COVID-19 admitted to Eka Kotebe General Hospital, Ethiopia, and conclude that ABO blood type is not associated with disease severity or mortality after multivariable adjustment [1]. We commend the authors for providing data from a low-resource setting, but we believe several aspects of design and analysis warrant clarification before inferring that ABO is irrelevant to COVID-19 outcomes.

A key issue is external validity and potential selection bias. In this cohort, blood group B was the most frequent (41.8%), whereas group O accounted for only 14.4% of patients. By contrast, contemporary Ethiopian blood-donor data from the Amhara region show group O to be predominant, followed by A, B and AB [2]. Such a shift in distribution may reflect regional variation, but it may also indicate that ABO type is already influencing the upstream risks of infection or hospital admission. The current analysis therefore mainly addresses prognosis among hospitalized patients and cannot exclude an association between ABO and the earlier stages of the disease pathway.

Another concern relates to statistical power and model specification. Only 49 patients had blood group AB and 82 had group O. These small cell counts, combined with adjustment for several comorbidities in multinomial and logistic regression models, are likely to produce wide confidence intervals that still encompass clinically relevant effects. A simple post-hoc calculation, using the reported sample sizes and plausible baseline risks of severe or critical COVID-19 of 20–40% in the reference category, suggests that the study has limited (well below 80%) power to detect odds ratios around 1.5 for group O versus non-O, with even lower power for group AB; only relatively large odds ratios (on the order of 2 or greater) would be detected with conventional power. Thus, the non-significant findings are compatible with a range of potentially important protective or harmful associations that this study could not reliably exclude. Moreover, low power in the presence of sparse data is not a neutral problem: in small samples, maximum likelihood estimates from logistic regression can be unstable, so that low power not only reduces the chance of detecting true effects but can also exaggerate the magnitude of estimated odds ratios or yield spurious “significant” associations [3,4]. Presenting absolute risks and confidence intervals by blood group, alongside adjusted measures of association (and, if feasible, more parsimonious or penalised models), would help readers judge whether the pattern of estimates is more consistent with true absence of effect or with imprecision and sparse-data bias.

A further consideration is residual confounding and effect modification. The models adjust for age, hypertension, diabetes and a composite comorbidity measure, but do not appear to incorporate sex, obesity, socio-economic factors, vaccination status or calendar period, all of which are determinants of both exposure and outcome in COVID-19 cohorts. Population-based data from Ontario show that, once vaccination and key covariates are accounted for, severe COVID-19 risk does not differ materially between O and non-O blood groups [5]. Conversely, an Iranian hospital cohort reported higher COVID-19 severity and mortality in A+ and lower risk in O+ patients [6]. Explicitly situating the Ethiopian findings within this heterogeneous literature would help clinicians understand whether the observed null association reflects biology, context or limitations of the dataset.

This study provides valuable information from an under-represented population. We suggest that clearer description of the source population and recruitment period, fuller reporting of effect estimates, and more detailed discussion of potential selection, sparse-data bias and residual confounding would strengthen the conclusions and better guide clinical interpretation and future research.

## Authors contribution

All authors contributed to the conception and drafting of this letter, critically appraised the cited studies, and approved the final version.

## CRediT authorship contribution statement

**Prashant Ramdas Kokiwar:** Writing – review & editing, Writing – original draft, Visualization, Validation, Supervision, Software, Resources, Project administration, Methodology, Investigation, Formal analysis, Data curation, Conceptualization. **Amit Singh Pawaiya:** Writing – review & editing, Writing – original draft, Visualization, Validation, Supervision, Software, Resources, Project administration, Methodology, Investigation, Formal analysis, Data curation, Conceptualization. **Ranjana Roy:** Writing – review & editing, Writing – original draft, Visualization, Validation, Supervision, Software, Resources, Project administration, Methodology, Investigation, Formal analysis, Data curation, Conceptualization. **Reenoo Jauhari:** Writing – review & editing, Writing – original draft, Visualization, Validation, Supervision, Software, Resources, Project administration, Methodology, Investigation, Formal analysis, Data curation, Conceptualization.

## Funding

No specific funding was received for this work.

## Declaration of competing interest

None declared.

## References

[1] Kitaw T.A., Haile R.N. (2025). Do certain blood groups increase COVID-19 severity and mortality?. Glob Epidemiol.

[2] Enawgaw B., Aynalem M., Melku M. (2022). Distribution of ABO and Rh-D blood group antigens among blood donors in the Amhara regional state. Ethiop J Blood Med.

[3] Greenland S., Mansournia M.A., Altman D.G. (2016). Sparse data bias: a problem hiding in plain sight. BMJ.

[4] Berkson J. (1955). Maximum likelihood and minimum | chi 2 estimates of the logistic function. J Am Stat Assoc.

[5] Ray J.G., Park A.L. (2022). SARS-CoV-2 vaccination, ABO blood group and risk of COVID-19: population-based cohort study. BMJ Open.

[6] Kouchek M., Miri M.M., Aghakhani K., Memarian A. (2024). ABO blood group is related to the prevalence, severity, and mortality rate of COVID-19. Med Clin Pract.

